# Clinical variables serve as prognostic factors in a model for survival from glioblastoma multiforme: an observational study of a cohort of consecutive non-selected patients from a single institution

**DOI:** 10.1186/1471-2407-13-402

**Published:** 2013-09-03

**Authors:** Signe Regner Michaelsen, Ib Jarle Christensen, Kirsten Grunnet, Marie-Thérése Stockhausen, Helle Broholm, Michael Kosteljanetz, Hans Skovgaard Poulsen

**Affiliations:** 1 Department of Radiation Biology, The Finsen Center, Copenhagen University Hospital, Blegdamsvej 9, DK-2100 Copenhagen, Denmark; 2 The Finsen Laboratory, Copenhagen University Hospital, Blegdamsvej 9, DK-2100 Copenhagen and Biotech Research and Innovation Center (BRIC), University of Copenhagen, Ole Maaløes Vej 5, DK-2200 Copenhagen, Denmark; 3 Department of Neuropathology, Center of Diagnostic Investigation, Copenhagen University Hospital, Blegdamsvej 9, DK-2100 Copenhagen, Denmark; 4 Department of Neurosurgery, The Neurocenter, Copenhagen University Hospital, Blegdamsvej 9, DK-2100 Copenhagen, Denmark

**Keywords:** Bevacizumab, Glioblastoma multiforme, Prognosis, Temozolomide

## Abstract

**Background:**

Although implementation of temozolomide (TMZ) as a part of primary therapy for glioblastoma multiforme (GBM) has resulted in improved patient survival, the disease is still incurable. Previous studies have correlated various parameters to survival, although no single parameter has yet been identified. More studies and new approaches to identify the best and worst performing patients are therefore in great demand.

**Methods:**

This study examined 225 consecutive, non-selected GBM patients with performance status (PS) 0–2 receiving postoperative radiotherapy with concomitant and adjuvant TMZ as primary therapy. At relapse, patients with PS 0–2 were mostly treated by reoperation and/or combination with bevacizumab/irinotecan (BEV/IRI), while a few received TMZ therapy if the recurrence-free period was >6 months.

**Results:**

Median overall survival and time to progression were 14.3 and 8.0 months, respectively. Second-line therapy indicated that reoperation and/or BEV/IRI increased patient survival compared with untreated patients and that BEV/IRI was more effective than reoperation alone. Patient age, ECOG PS, and use of corticosteroid therapy were significantly correlated with patient survival and disease progression on univariate analysis, whereas p53, epidermal growth factor receptor, and O^6^-methylguanine-DNA methyltransferase expression (all detected by immunohistochemistry), tumor size or multifocality, and extent of primary operation were not. A model based on age, ECOG PS, and corticosteroids use was able to predict survival probability for an individual patient.

**Conclusion:**

The survival of RT/TMZ-treated GBM patients can be predicted based on patient age, ECOG PS, and corticosteroid therapy status.

## Background

Glioblastoma multiforme (GBM) is the most common adult primary brain tumor [1] and patients generally have a dismal prognosis with a median survival of just 15 months [2]. Newly diagnosed patients often undergo surgical tumor resection and studies have shown that the extent of surgical resection is correlated with increased median survival duration [3,4]. Given that surgery as a single treatment is insufficient due to a diffuse infiltration by tumor tissue into the brain, patients generally receive concomitant and adjuvant chemotherapy with temozolomide (TMZ) in combination with radiotherapy (RT) [2,5]. TMZ is an alkylating agent that induces cell death primarily through the formation of O^6^-methylguanine DNA adducts, resulting in DNA double-strand breaks [6]. The drug is well tolerated with mostly mild to moderate adverse events [7]. Preclinical studies have shown that TMZ sensitizes GBM cells to RT [8,9], which might explain why the combination is favorable. However, despite the EORTC-NCIC trial originally showing a survival benefit in all patients treated with RT/TMZ, 5-year follow-up analysis showed that nearly all patients experienced relapse and only 9.8% survived 5 years after initial diagnosis [10].

The response to and survival following RT/TMZ therapy has been correlated with several patient-specific variables. The most frequently reported predictive variables include patient age, performance status (PS), extent of surgical resection, and expression of O^6^-methylguanine-DNA methyltransferase (MGMT) [11-16], a DNA repair protein inhibiting the effect of TMZ by reversing alkylation [17]. Predictive variables that have been less frequently reported include tumor size [12], corticosteroid therapy [14], and positivity for a number of overexpressed or mutated molecules, including epidermal growth factor receptor (EGFR), and p53 [18,19].

Although GBM tends to recur locally [20], repeat surgery is only a treatment option for a limited number of patients due to poor PS, large tumor volumes, and involvement of critical brain areas [21]. As an alternative, patients with relapsed tumors have received chemotherapy or different kinds of molecular-targeted therapies [5]. Among the latter is bevacizumab (BEV), a humanized monoclonal antibody targeting vascular endothelial growth factor (VEGF). VEGF promotes proliferation, survival, and migration of endothelial cells, and is expressed and released mainly from tumor cells in response to pro-angiogenic stimuli [22]. GBM is one of the most vascularized tumors [23] and GBM tumors express high levels of angiogenic factors including VEGF [24]. Various studies, both retrospective and prospective, have shown that BEV with or without cytotoxic chemotherapy results in a substantive response rate and improved 6-month progression-free survival in GBM patients who have relapsed after previous RT/TMZ treatment [25]. However, the effect of BEV on overall survival (OS) has been somewhat modest, with most studies reporting median OS values of <10 months after initiation of BEV therapy [25].

To maximize patient survival and avoid unnecessary treatments, prognostic parameters must be taken into account when deciding which treatment modality is most appropriate for the individual patient. Recursive partitioning analysis (RPA) is a tool developed in the early 1990s with which it is possible to categorize brain cancer patients into subgroups with different median survival according to a number of clinical and therapeutic parameters [26]. GBM-specific adaptations have been developed [27,28] and research has shown prognostic significance of the classification for GBM patients receiving RT with or without TMZ [27]. However, the RPA classification is somewhat crude as a prognostic tool for therapeutic decision making and is more useful for the stratification of patients in clinical trials. As an alternative, more precise prognostic calculators have been developed for GBM patients receiving RT/TMZ [14,29]. However, as the number of studies of prognostic calculators in GBM patients is limited, the approach needs further investigation.

In this study we analyzed clinical and molecular data retrospectively in a cohort of 225 newly diagnosed consecutive GBM patients treated with RT/TMZ as primary therapy. Parameters identified to correlate to tumor progression and patient survival were assembled in a prognostic model able to predict patient survival. In addition, the effects of repeat surgery, BEV plus irinotecan (IRI) therapy, and the combination of both therapeutic modalities were compared for the treatment of relapsed tumors.

## Methods

This study was performed according to the Declaration of Helsinki and Danish legislation. Permissions were given from the Danish Data Protection Agency (2006-41-6979) and the ethical committee for the Capital Region of Denmark (H-C-2008-095).

### Patients

This study included a consecutive series of 225 patients with newly diagnosed GBM (WHO grade IV) recruited from 2005 to 2010 who were not selected other than having ECOG PS 0–2. There were 80 women and 145 men with a median age of 59.2 years (range, 22.6–75.4 years). Of these, 198 patients presented with a single tumor, while 26 patients had multifocal disease (data missing, *n* = 1). ECOG PS was 0 (*n* = 132), 1 (*n* = 66), or 2 (*n* = 19) [data missing, *n* = 8]. Patient demographics are shown in Table 1.

**Table 1:** **Patient demographics, therapy, and response (*N*** **= 225)**

	
Age (years), median (range)	59.2 (22.6–75.4)
Gender, *n* (%)	
Female	80 (35.6)
Male	145 (64.4)
ECOG performance status, *n* (%)	
0	132 (58.7)
1	66 (29.3)
2	19 (8.4)
Missing	8 (3.6)
Multifocal Disease, *n* (%)	
Yes	26 (11.6)
No	198 (88)
Missing	1 (0.4)
Extent of tumor resection, *n* (%)	
Biopsy	29 (12.9)
Partial resection	104 (46.2)
Gross total resection	89 (39.6)
Missing	3 (1.3)
Corticosteroid therapy at initiation of RT/TMZ, *n* (%)	
Yes	165 (73.3)
No	57 (25.3)
Missing	3 (1.3)
No. of TMZ cycles following initial RT/TMZ, *n* (%)	
Median	3
0	36 (16.0)
1	13 (5.8)
2	54 (24.0)
3	12 (5.3)
4	10 (4.4)
5	23 (10.2)
6	75 (33.3)
Missing	2 (0.9)
Reoperation, *n* (%)	
Yes	74 (33.0)
No	151 (67.0)
Second-line TMZ therapy, *n* (%)	
Yes	12 (5.3)
No	213 (94.7)
Second-line BEV/IRI therapy	
Yes	85 (37.8)
No	132(58.7)
Missing	8 (3.6)
Follow-up duration (months), median (range)	60 (23–92)
Best clinical response, *n* (%)	
CR	6 (2.7)
PR	17 (7.5)
SD	93 (41.3)
PD	94 (41.8)
Missing	15 (6.7)

*Abbreviations: CR* complete response, *PD* progressive disease, *PR* partial response, *RT* radiotherapy, *SD* stable disease, *TMZ* temozolomide.

### Treatments

Patients underwent surgery, taking either a tumor biopsy (*n* = 29) or resulting in partial (*n* = 104) or complete tumor resection (*n* = 89) prior to additional therapy (data missing, *n* = 3). The extent of surgical radicality was based on the impression of the surgeon.

Patients received 6 weeks of concomitant RT/TMZ therapy as primary treatment. They received TMZ 75 mg/m^2^/day plus RT at a dose of 60 Gy to the planning target volume in 30 fractions with 5 fractions/week delivered by a megavoltage linear accelerator. Cerebral CT was performed with 3-mm slices and fused with baseline MRI for treatment planning. Treatment planning was performed three-dimensionally using Eclipse™ treatment planning system (Varian Medical Systems, Palo Alto, CA) and volumes of interest were defined in agreement with International Commission on Radiation Units & Measurements Reports 50 and 62. The gross tumor volume (GTV) was defined as the contrast-enhanced tumor on post-contrast T1 image and/or the non-enhancing area on the T2 image on the baseline MRI scan. The clinical target volume (CTV) as defined as the GTV + 2 cm margin, except for bony structures. Meningeal structures were considered anatomic barriers to tumor spread, if appropriate clinically. If present, the surgical cavity was included. The internal target volume was identical to the CTV. No variations in size, shape or position of CTV in relation to anatomical reference structures were considered. Planning target volume was defined as the CTV + 0.5 cm margin for patient setup inconsistencies. Tolerance doses for organs at risk were as described by Emami *et al*. [30].

During this treatment, patients were also given antibiotic prophylaxis with 400 mg sulfamethoxazole/80 mg trimethoprim 3 times/week. In addition, a number of patients received corticosteroid therapy to relieve neurological symptoms: 165 patients (73%) received corticosteroid therapy at the initiation of RT/TMZ therapy.

Four weeks after completion of initial therapy, patients were given up to six courses of adjuvant TMZ therapy, with one course defined as TMZ for 5 days followed by 23 days without therapy. The initial course was given at a dose of 150 mg/m^2^/day and the remaining courses at a dose of 200 mg/m^2^/day. The dose was adjusted based on relevant blood tests. The number of adjuvant TMZ therapy courses given is summarized in Table 1.

As therapy for recurrent tumors, patients who maintained ECOG PS 0–2 were initially considered for secondary surgery to remove as much tumor as possible. These patients were thereafter considered for secondary therapy with TMZ 150–200 mg/m^2^/day if they had already received 6 courses of adjuvant TMZ and thereafter had a recurrence-free period ≥6 months. The courses consisted of 5 days TMZ therapy followed by 23 days without therapy. From 2006, regardless of adjuvant TMZ therapy and extent of recurrence-free period, the patients were additionally considered for second-line therapy with BEV 10 mg/kg every 2 weeks and irinotecan (IRI), as previously described [31]. In total, 74 patients underwent secondary surgery, 12 received second-line therapy with TMZ, and 85 received second-line therapy with BEV/IRI. Characterization of the therapy is detailed in Table 1.

### Clinical evaluation

At treatment initiation, a full medical history was determined and patients were examined for baseline physical and neurological status. In addition, ECOG PS [32] was determined, routine laboratory tests (including blood chemistry and urinalysis) were performed, and MRI scans were undertaken to evaluate tumor size and location.

The median duration of observation from the day patients first received therapy to the project cut-off day (22 October 2012) was 60 months (range, 23–92 months). In this period contrast and non-contrast MRI scans were repeated after 2, 5, and 6 courses of adjuvant TMZ. Patients’ neurological and clinical performance, together with corticosteroid treatment, was recorded at these time points. All patients were thereafter followed every 3 months until death or study cut-off date using the same procedures. Safety was determined using NCI-CTCAE, version 3.0, criteria [33].

### Histological and immunohistochemical evaluation

**Figure 1:**
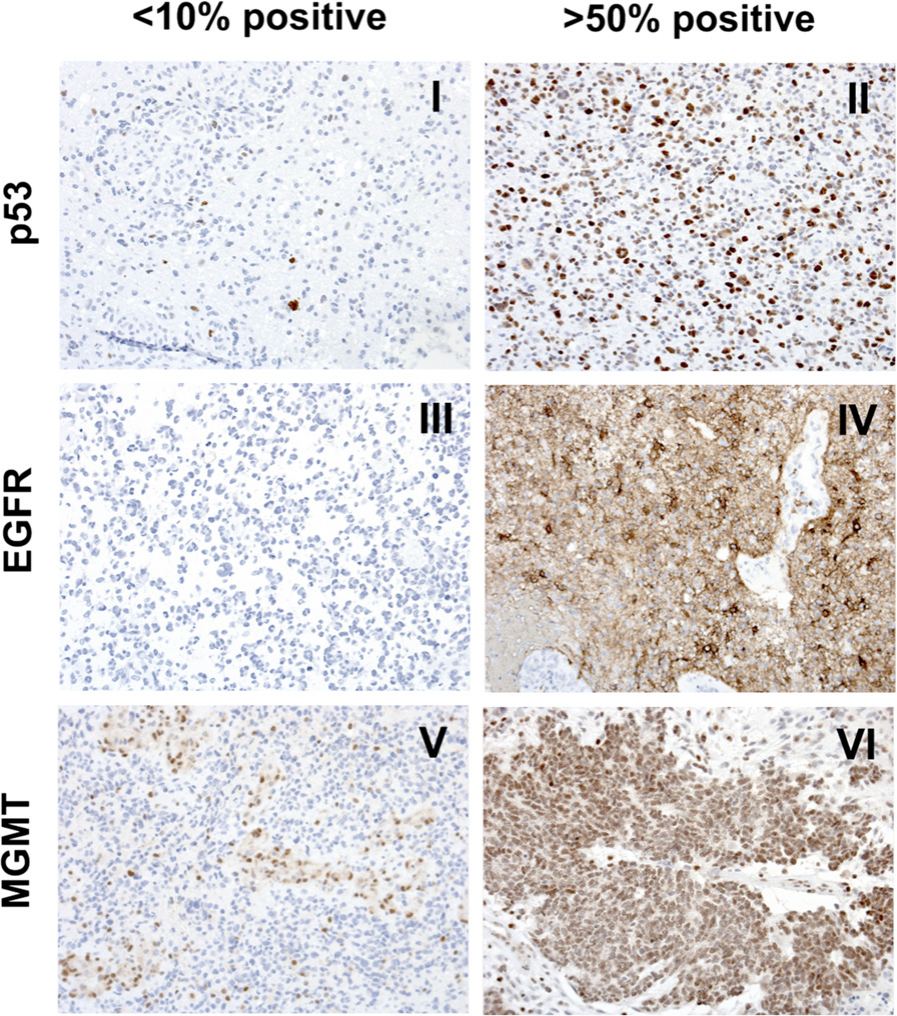
**Examples of IHC stainings.** p53, EGFR, and MGMT expression by IHC scored semiquantitatively on the scale: <10%, 10–25%, 25–50%, and >50% cells stained positive. Representative photomicrographs (200× magnification) are given for GBM tissue with <10% (I, III, V) and >50% (II, IV, VI) positive cells. Cells staining positive for MGMT in V represent macrophages and endothelial cells of vessels, while it is tumor cells that stains positive for p53 in I.

Evaluations were made on formalin-fixed, paraffin-embedded tissue. Tumor tissue was classified and graded as GBM according to WHO 2007 guidelines. Diagnosis was based on conventional histological and immunohistochemical (IHC) procedures, including staining with hematoxylin and eosin, glial fibrillary acidic protein (GFAP), p53, EGFR, and MGMT. For IHC, sections were pre-treated in a microwave oven with a Tris/ethylene glycol tetra-acetic acid buffer (pH 9.0) and immunostained on a DAKO Cytomation autostainer using murine monoclonal antihuman antibodies against GFAP (Z 0334, 1:6400), p53 (M 7001, 1:800), EGFR (M 7239, 1:200) [all from DAKO, Glostrup, Denmark] and MGMT (MAB16200, 1:200, Millipore, USA). The p53, EGFR, and MGMT IHC reactions were semiquantitatively evaluated according to the number of cells stained: <10%, 10–25%, 26–50%, and >50%. Staining examples are shown in Figure 1. For statistical analysis, expression evaluated as <10% was considered negative, while ≥10% was considered positive.

### Study endpoints

Study endpoints were time to progression (TTP), OS, OS from recurrence, response at 3 and 6 months, and best response. TTP was defined as the time from the start of RT/TMZ treatment to radiological or clinical progression. OS was defined as the time from start of RT/TMZ treatment until death from any cause, while OS from recurrence was defined as the time from tumor relapse until death from any cause.

Response was evaluated 3 and 6 months after completion of RT. Response evaluation was based on MacDonald criteria [34], considering MRI measurements of contrast-enhancing tumor size and recording of the largest cross-sectional area of the tumor, patient neurological status, and corticosteroid dose. Complete response (CR) was defined as complete disappearance of measurable disease by MRI, partial response (PR) as >50% reduction of MRI contrast enhancing tumor, and progressive disease (PD) as >25% increase in area of contrast enhancement. Patients with CR or PR also had to be taking the same or decreased corticosteroid dose and have stable or improved neurological findings. Patients, by definition, had stable disease (SD) if the criteria for CR, PR, or PD were not met and no clinical progression was observed.

For each patient, responses after 3 and 6 months were compared and a ‘best response’ determined, defined as the maximum achieved response registered for the patient in the observation period.

### Statistical considerations

Factors that were analyzed as potential markers of prognostic significance included: age, gender, ECOG PS, extent of resection, tumor location, tumor size, previous corticosteroid therapy, and tumor EGFR, p53, and MGMT expression. Univariate and multivariate analyses of response data were performed using logistic regression analysis modeling the probability of MacDonald response at 3 and 6 months as well as the best response. Estimates of survival probabilities for OS (primary endpoint) and TTP (secondary endpoint) were performed by the Kaplan-Meier method. Univariate and multivariate analyses of OS and TTP for the chosen explanatory variables were performed using the Cox proportional hazards regression model. Analysis of time-dependent variables was performed using the landmark method as well as the time-dependent Cox regression model.

The final model was chosen using a backwards selection procedure, the entry level was 5%. The analysis was repeated removing the least significant covariate in order to use all available data, in particular the molecular markers were only done for a subset of patients. Model assessment was done using Schoenfeld and martingale residuals. The overall concordance index (C-index) was used as a measure of discrimination [35,36] and calculated in accordance to previously published guidelines [37]. In addition, a 5 fold cross validation was done to evaluate the model.

*P* values < .05 were considered significant. Calculations have been performed using IBM SPSS Statistics (v19.0, IBM Denmark, Kgs. Lyngby, Denmark) and SAS (v9.2, SAS Institute, Cary, NC) software.

## Results

### Significant factors affecting outcome from first-line RT/TMZ

As shown in Table 1, best responses to first-line RT/TMZ among evaluable patients were: CR (*n* = 6; 2.9%); PR (*n* = 17; 8.1%); SD (*n* = 93; 44.3%); and PD (*n* = 94; 44.8%). Data were missing for 15 patients, who were therefore not evaluated. The effects of clinical and molecular variables on best response and response at 3 and 6 months on univariate analysis are summarized in Tables 2 and 3, respectively. The only clinical variable with a significant effect on response was patient age, for which a 10-year increase resulted in a reduction of the best response (*P* = .045). None of the other clinical factors examined had a statistically significant impact on patient best response or response at 3 and 6 months. EGFR, p53, and MGMT expression were examined as potential molecular markers for response (Table 3). Because of missing data, analyses were only available for subsets of patients: 145 of 199 patients presented EGFR-positive tumors; 105 of 202 patients presented p53-positive tumors; and 65 of 163 patients presented MGMT-positive tumors. There was no significant correlation between EGFR and MGMT expression and best response or response at 3 and 6 months. The odds ratio for response at 3 months among patients with p53-positive tumors was significantly (*P* = .043) higher as compared to those with p53-negative tumors. Although not significant, this tendency was also seen for the best response (*P* = .053) but not for response at 6 months (*P* = .21).

**Table 2:** Univariate analysis of correlation of clinical variables with survival, disease progression, and response

**Covariate**	**OS**	**TTP**	**OS from recurrence**	**Response at 3 months**	**Response at 6 months**	**Best response**
	**(HR) [95% CI]**	**(HR) [95% CI]**	**(HR) [95% CI]**	**(OR) [95% CI]**	**(OR) [95% CI]**	**(OR) [95% CI]**
Operation						
Gross total *vs.* biopsy	0.76 (0.49–1.17)	0.74 (0.48–1.16)	0.81 (0.51–1.27)	4.33 (0.54–35)	1.88 (0.21–16.9)	4.00 (0.49–32)
Partial *vs.* biopsy	0.98 (0.64–1.50)	0.86 (0.56–1.32)	1.05 (0.68–1.65)	1.37 (0.15–12.2)	0.86 (0.09–8.22)	2.13 (0.25–17)
	*P* = .22	*P* = .37	*P* = .23	*P* = .07	*P* = .35	*P* = .24
Age (per 10-year increase)	1.36 (1.17–1.58)	1.17 (1.01–1.36)	1.36 (1.16–1.60)	0.66 (0.43–1.01)	0.76 (0.46–1.23)	0.66 (0.44–0.99)
	*P* < .0001	*P* = .034	*P* = .0001	*P* = .056	*P* = .26	*P* = .045
Gender (female *vs.* male)	1.11 (0.83–1.47)	1.07 (0.8–1.44)	1.01 (0.75–1.37)	1.48 (0.59–3.75)	1.31 (0.49–3.53)	1.68 (0.70–4.02)
	*P* = .47	*P* = .64	*P* = .94	*P* = .41	*P* = .29	*P* = .24
Multifocal *vs.* single lesion	1.23 (0.80–1.88)	1.26 (0.82–1.93)	1.16 (0.74–1.81)	NA	NA	NA
	*P* = .34	*P* = .29	*P* = .52			
Tumor size (2-fold increase)	1.00 (0.88–1.14)	0.98 (0.87–1.11)	1.03 (0.90–1.19)	1.41 (0.87–2.29)	1.56 (0.95–2.57)	1.39 (0.89–2.16)
	*P* = .97	*P* = .74	*P* = .64	*P* = .16	*P* = .08	*P* = .15
Corticosteroid therapy (yes *vs.* no)	2.13 (1.49–2.86)	1.41 (1.02–1.92)	2.17 (1.54–3.03)	0.44 (0.17–1.11)	0.78 (0.28–2.17)	0.57 (0.23–1.39)
	*P* < .0001	*P* = .036	*P* < .0001	*P* = .08	*P* = .63	*P* = .22
ECOG performance status						
1 *vs.* 0	1.42 (1.04–1.94)	1.33 (0.97–1.84)	1.58 (1.13–2.20)	0.26 (0.06–1.16)	0.22 (0.05–0.97)	0.22(0.05–0.97)
2 *vs.* 0	2.31 (1.40–3.82)	1.70 (1.03–2.80)	2.34 (1.39–3.93)	0.88 (0.18–4.19)	0.71 (0.15–3.32)	0.71(0.15–3.35)
	*P* = .0015	*P* = .046	*P* = .0007	*P* = .21	*P* = .13	*P* = .13

*Abbreviations: CI* confidence interval, *HR* hazard ratio, *NA* not analyzed, *OR* odds ratio, *OS* overall survival, *TTP* time to disease progression.

**Table 3:** Univariate analysis of correlation of molecular markers with survival, disease progression, and response

**Covariate**	**OS**	**TTP**	**OS from recurrence**	**Response at 3 months**	**Response at 6 months**	**Best response**
	**(HR) [95% CI]**	**(HR) [95% CI]**	**(HR) [95% CI]**	**(OR) [95% CI]**	**(OR) [95% CI]**	**(OR) [95% CI]**
EGFR						
Positive (*n* = 145)	1.05 (0.77-1.43)	0.82 (0.61-1.12)	1.02 (0.75-1.41)	0.64 (0.22-1.86)	0.64 (0.46-4.15)	1.06 (0.22-1.91)
	*P* = .75	*P* = .21	*P* = .89	*P* = .41	*P* = .96	*P* = .43
Negative (*n* = 54)						
Missing (*n* = 26)						
p53						
Positive (*n* = 105)	0.76 (0.55-1.05)	0.92 (0.66-1.27)	0.73 (0.52-1.03)	3.01 (1.04-8.7)	2.04 (0.68-6.1)	2.64 (0.99-7.1)
	*P* = 0.10	*P* = .60	*P* = .071	*P* = .043	*P* = .21	*P* = .053
Negative (*n* = 97)						
Missing (*n* = 23)						
MGMT						
Positive (*n* = 65)	0.97 (0.64-1.48)	0.90 (0.59-1.36)	1.42 (0.91-2.19)	1.36 (0.35-5.34)	1.02 (0.25-4.18)	1.78 (0.52-6.13)
	*P* = 0.89	*P* = 0.61	*P* = .12	*P* = 0.66	*P* = .98	*P* = .36
Negative (*n* = 98)						
Missing (*n* = 62)						

Positive and negative expression are defined by ≥10% and <10% of cells, respectively, stained on immunohistochemical analysis. *Abbreviations: CI* confidence interval, *HR* hazard ratio, *OR* odds ratio, *OS* overall survival, *TTP* time to disease progression.

**Figure 2:**
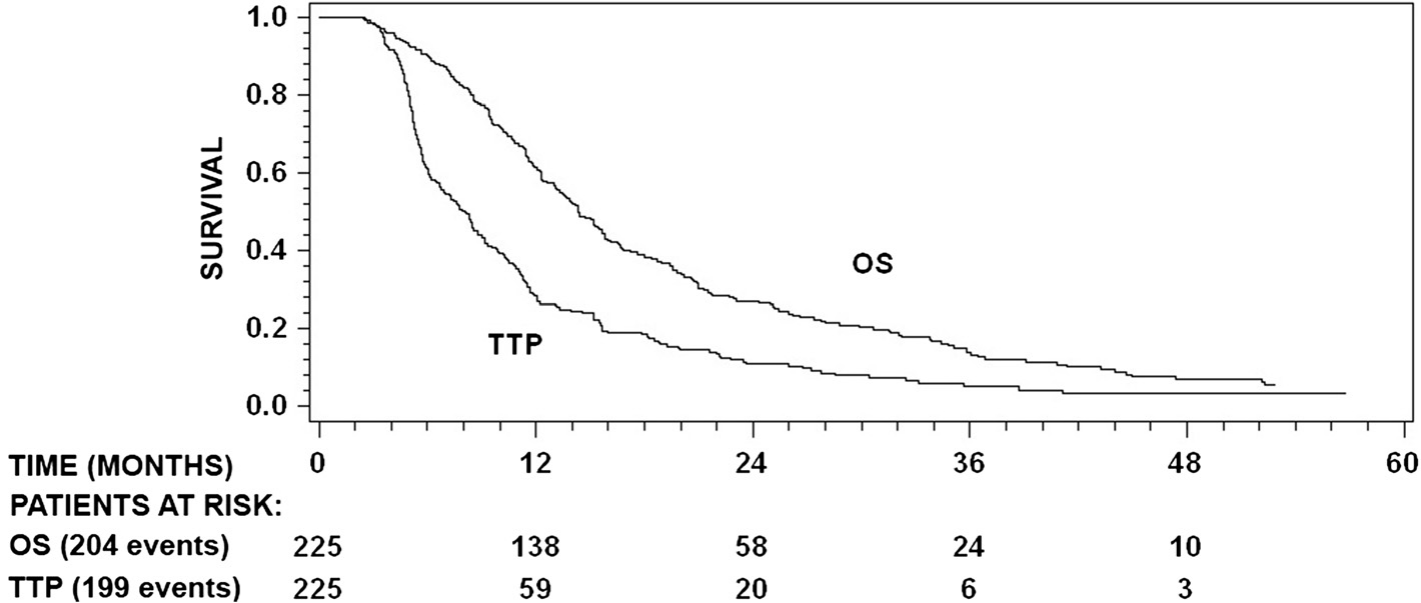
**Kaplan-Meier plots showing TTP and OS for the patient population.** The curves are based on data from all 225 examined patients. Numbers for patients at risk at selected times are shown in addition to the total number of events (deaths for OS and progression for TTP).

All 225 patients had TTP data, of whom 199 had disease progression. Median TTP was 8.0 months (95% CI, 6.7–9.0 months) with progression free survival of 61% (95% CI, 54–67%) at 6 months and 28% (95% CI, 22–34%) at 12 months (Figure 2). Increased patient age (*P* = .034), higher ECOG PS score (*P* = .046), and use of corticosteroid therapy at RT/TMZ initiation (*P* = .036) had a significant negative impact on TTP (Table 2). None of the other examined clinical or molecular variables had a significant impact on TTP (Tables 2 and 3).

All 225 patients had OS data, of whom 204 (90.7%) died during the observation period. Median OS was 14.3 months (95% CI, 13.0–15.8 months) with an OS rate of 27.1% (95% CI, 21–33%) at 2 years and 13.9% (95% CI, 9.5–19.0%) at 3 years (Figure 2). Median OS from tumor recurrence was 5.9 months (95% CI, 5.0–6.9 months). Increased patient age (*P* < .0001), higher ECOG PS score (*P* = .0015), and use of corticosteroid therapy at RT/TMZ initiation (*P* < .0001) had a significant negative impact on OS (Table 2). Increased patient age (*P* = .0001), higher ECOG PS score (*P* = .0007), and use of corticosteroid therapy at RT/TMZ initiation (*P* < .0001) also showed a significant negative correlation with decreased OS from disease recurrence. None of the other clinical covariates were significantly correlated with OS or OS from disease recurrence. None of the molecular markers (EGFR, p53, and MGMT) were significantly correlated with patient survival (Table 3). There was a non-significant trend for longer OS (*P* = .10) and OS from disease recurrence (*P* = .071) among patients with p53-positive tumors as compared to those with p53-negative tumors.

### Reoperation and second-line BEV/IRI therapy for relapsed-tumors improve survival

A total of 199 patients presented relapse. Most of these patients underwent reoperation of the tumor (*n* = 31; 15.6%), received BEV/IRI therapy (*n* = 42; 21.1%), or had a combination of both modalities (*n* = 43; 21.6%) for recurrent disease. In addition, 12 patients received second-line TMZ therapy as they had received 6 courses of adjuvant TMZ therapy and did not have disease recurrence for >6 months: due to the limited number of patients receiving this therapeutic option, this treatment was excluded when analyzing the effect of the different second-line treatments on survival. Compared to patients who received no second-line therapy, there was a significant OS increase in those who underwent reoperation (hazard ratio (HR) = 0.39; 95% CI, 0.25–0.60) or received BEV/IRI therapy (HR = 0.23; 95% CI, 0.15–0.34) as single treatments. When comparing OS for patients who received BEV/IRI as single second-line therapy with those who received a combination of reoperation plus second-line BEV/IRI therapy, there was no significant beneficial effect, although there was a tendency for better survival among those who received the combination (HR = 0.87; 95% CI, 0.46– 1.37). In contrast, when the reoperation-BEV/IRI combination was compared to reoperation alone, there was a significant increase in survival (HR = 0.51; 95% CI, 0.31–0.83).

### A prognostic model can predict survival of GBM patients receiving RT/TMZ

Multivariable analyses of TTP and OS were done including the covariates described in Tables 2 and 3.

Multivariable analysis of the secondary endpoint, TTP, yielded a final model only including corticosteroid therapy (yes vs no, HR = 1.41 (95% CI, 1.02-1.92), p = 0.036). The p-values to include ECOG PS and age in the final model were 0.12 and 0.21 respectively. The p-values to include the remaining covariates were all >0.14.

A final model was selected for the primary endpoint OS, the following covariates were statistically significant ECOG PS (PS 1 vs 0, HR = 1.22 (95% CI, 0.89-1.68), PS 2 vs 0, HR = 2.06 (95% CI, 1.25-3.42), p = 0.015), corticosteroid therapy (yes vs no, HR = 2.06 (95% CI, 1.47-2.87), p < 0.0001) and age (per 10 years, HR = 1.31 (95% CI, 1.11-1.54), p = 0.001). P values to include the excluded covariates in the final model were >0.17 (P53, p = 0.57; MGMT, p = 0.24; EGFR, p = 0.45; tumor size, p = 0.51; operation, p = 0.84; multifocal *vs.* single lesion, p = 0.17). Significant interactions could not be demonstrated suggesting an additive effect of these covariates. Model assessment was found to adequate. The overall concordance index (C-index) [35-37] for the final model was 0.82 (95% CI, 0.71–0.92), which can be interpreted as the probability of concordance between predicted and observed survival, thereby demonstrating a substantial discrimination for this model. The results of the five-fold internal cross validation supported the chosen model validating the model in the 5 test sets (C-indices > 0.80).

Based on estimated regression coefficients, patient survival chances at 6, 12, 18, and 24 months after diagnosis were calculated for various levels of each of the three covariates (Table 4). For example, the survival probability for a 40-year-old patient with ECOG PS 0 receiving no corticosteroid therapy was 97%, 86%, 73%, and 64% at 6, 12, 18 and 24 months, respectively, following diagnosis. A much lower survival probability is exemplified for a 80-year-old patient with ECOG PS 2 receiving corticosteroids: 67%, 15%, 2%, and 0% at 6, 12, 18, and 24 months, respectively from diagnosis. It can also be seen that a change in several variables at the same time can have a major negative impact on the survival probability for the individual patient, while a change in only one of the three factors had a relatively minor impact on the survival probability. This is exemplified by a survival probability of 24% at 12 months after diagnosis for a 70-year-old patient with ECOG PS 2 receiving corticosteroid therapy compared to 67% for a 50-year-old patient with ECOG PS 0 receiving corticosteroid therapy, and 82% for a 50-year-old patient with ECOG PS 0 not receiving corticosteroid therapy. It is noteworthy that a 20-year increase of patient age has a negative effect on survival probability that is similar to that seen for an increase in ECOG PS from 0 to 2 or corticosteroid therapy *vs.* no therapy.

**Table 4:** Estimated survival probabilities from diagnosis depending on patient ECOG PS, corticosteroid therapy use and age

**ECOG PS**	**Steroid therapy**	**Age (years)**	**Survival probability (%)**			
			**At 6 months**	**At 12 months**	**At 18 months**	**At 24 months**
0	No	40	97	86	73	64
		45	96	84	70	60
		50	96	82	66	56
		55	95	80	62	51
		60	95	77	58	46
		65	94	74	54	41
		70	93	71	49	36
		75	92	68	44	32
		80	91	64	39	27
	Yes	40	94	73	52	40
		45	93	70	48	35
		50	92	67	43	30
		55	91	63	38	25
		60	89	59	33	21
		65	88	54	28	16
		70	86	50	23	13
		75	84	45	19	9
		80	82	40	15	7
1	No	40	96	83	68	58
		45	96	81	64	53
		50	95	79	60	49
		55	94	76	56	44
		60	93	73	52	39
		65	93	70	47	34
		70	91	66	42	29
		75	90	62	37	24
		80	89	58	32	20
	Yes	40	92	68	45	32
		45	91	65	40	28
		50	90	61	35	23
		55	89	57	30	18
		60	87	52	26	14
		65	85	47	21	11
		70	83	43	17	8
		75	81	38	13	5
		80	79	33	10	4
2	No	40	94	73	52	40
		45	93	70	47	35
		50	92	67	43	30
		55	90	63	38	25
		60	89	59	33	20
		65	88	54	28	16
		70	86	50	23	12
		75	84	45	19	9
		80	82	40	15	7
	Yes	40	87	53	26	15
		45	85	48	22	11
		50	84	43	17	8
		55	81	38	13	6
		60	79	33	10	4
		65	76	28	7	2
		70	73	24	5	1
		75	70	19	3	1
		80	67	15	2	0

## Discussion

In this study, we examined a cohort of newly diagnosed GBM patients treated with RT plus concomitant and adjuvant TMZ as primary therapy. We observed a median OS of 14.3 months and a median TTP of 8.0 months (Figure 2), which is very similar to values found in the EORTC-NCIC trial (14.6 and 6.9 months respectively) [2]. Based on this and the fact that the examined patients were consecutive and not selected, we conclude that the patients included are good representatives for the general population affected with GBM.

As treatment for recurrent disease, we found that both BEV/IRI therapy and reoperation resulted in significantly increased OS compared to untreated patients, which is in line with other studies [25,38]. In addition, our results indicate that BEV/IRI therapy is more effective than reoperation as second-line therapy for the majority of patients with recurrent GBM tumors and that the therapy should be given in combination with reoperation when possible. However, as the second-line treatments were based on individual evaluation of patient health status and not on a randomized trial, this could result from the fact that mainly the best performing patients received the reoperation and BEV/IRI combination. Randomized clinical trials are therefore needed for a better comparison of these two different second-line treatments.

Although RT/TMZ improves survival as compared to patients receiving RT alone, it only results in long-term survival (>2 years) for <30% of patients [10]. Much effort has been devoted to finding parameters that correlate with response to and survival following RT/TMZ therapy. Using univariate analysis in the present study, we found that three clinical markers (age, ECOG PS, and corticosteroid therapy at treatment initiation) had a significant impact on survival following therapy (Table 2). All three variables have been previously reported to affect survival. However, while an analysis of the EORTC-NCIC trial data was able to find an impact of all three variables [14], studies on other patient groups only saw a significant effect for one of these markers [12,16].

Contrary to our expatiations, we were not able to find any significance from the extent of primary operation in our study (Table 2), although several other studies have shown a significant effect for this variable on the response and survival of GBM patients treated with RT/TMZ [13-15,39]. As in other studies with similar negative results [12,40], we expect that the non-significant result is caused by incorrect assessment of surgical radicality, which in this study was estimated based on surgeons’ impression of tumor remaining in the resection area. Supporting this is the significant effect observed in our study for second-line reoperation, which was performed by a more experienced team of surgeons at our institution. Our results underline the importance of standardizing the evaluation process, in which the use of early (within 72 hours of surgery) MRI scans could be an important tool, a method used in several studies finding an effect of primary surgery [13,39].

There is a strong indication for the involvement of EGFR and p53 in the response of GBM to TMZ. Studies on GBM cells couple signaling from the EGFR receptor to reduced sensitivity to chemotherapeutic agents that, like TMZ, have alkylating activity [41,42], while p53 inactivation in GBM cells results in increased TMZ sensitivity [43,44]. However, in line with previous studies examining the prognostic value of EGFR [13,18,40,45] in TMZ-treated GBM patients, we were unable to find a significant correlation between this molecule and patient response or survival (Table 3). We found a significantly increased response rate in patients who had p53-positive tumors compared to those with p53-negative tumors, although we were unable to find a significant effect on OS and TTP. This adds to the conflicting picture existing for this molecule, for which both significant and non-significant results exist regarding its effect on response and survival [13,18,40,45]. Overall, these results indicate that EGFR and p53, despite their involvement in GBM tumor development and growth, not are main players in the response of GBM tumors to TMZ. However, improved assay techniques and consideration of tumor heterogeneity are necessary to confirm this.

Many studies have shown a significant correlation between lack of MGMT expression and survival of TMZ-treated GBM patients [11,13,14]. However, the detection method varies from direct detection of the MGMT protein to indirect detection of the methylation status of the MGMT promoter as a marker for its expression [46]. In line with previous studies [12,13], we were unable to show a significant correlation between MGMT status and outcome following RT/TMZ therapy when detecting MGMT at the protein level using IHC. This, combined with an analysis which found that MGMT protein expression does not correlate with the promoter methylation status of MGMT [47], indicates that IHC is not a reliable technique for MGMT detection for prediction of patient response to TMZ.

Emerging results show that GBM tumors can be subclassified into different groups based on their molecular expression patterns and that these subclasses correlate to variations in patient survival [48,49]. This observation indicates that individualized therapy could be a way to increase the survival of GBM patients.

Research conducted on parameters that are able to predict response and survival following TMZ therapy has mostly centered on single markers. This has resulted in the identification of a number of both clinical and molecular parameters [11-13,16], but none of these have been able to give an accurate prediction of RT/TMZ therapy outcome for the individual patient. As a result, no markers have been implemented to segregate patients into responders and non-responders for RT/TMZ therapy, although the combination has been given as standard therapy for GBM patients since 2005. That an approach taking several markers into account simultaneously is beneficial is indicated by the ability of the RPA classification system to subgroup RT/TMZ-treated patients according to survival [27] and by studies that are able to increase the predictive effect using multigene [50] or multimethylation [51] profiles as compared to the use of single variables.

Based on these facts, we assembled a model to predict patient survival using the individual variables that we had identified as significant for survival (age, ECOG PS and corticosteroid therapy at treatment initiation). The model, which was developed using cox modelling, is able to calculate the probability for a given patient receiving the described therapy to be alive at a given time and can be used to identify patients with the best and worst survival chances (Table 4). Another approach could be recursive partitioning, thereby making a decision tree model as used in the RPA classification system. However, as discussed previously [14,28], this approach groups the variables into only a few categories and cannot predict the survival for the individual patient.

Furthermore, our model contributes to the debate on which therapeutic option should be preferred for elderly patients [52]. Both RT [53] and TMZ [54] have been proven to result in survival benefit for elderly GBM patients. However, due to the general belief that elderly patients do not tolerate concomitant chemo-radiotherapy as well as younger patients in combination with the observation of a negative correlation between patient age and the survival following RT/TMZ therapy [14], this combination is not standard in elderly patients. Our results indicate that age alone should not disqualify patients from concomitant RT/TMZ therapy, but that ECOG PS and use of corticosteroid therapy should be taken into account for making any therapeutic decisions. This conclusion supports several studies which have found that RT/TMZ therapy is effective in elderly GBM patients presenting with good prognostic factors [15,55].

A few other studies have constructed prognostic models for GBM patients. One model established from patients receiving RT/TMZ as primary treatment in the EORTC-NCIC trials includes age, PS, MGMT status, extent of resection, and mental state [14]. Another model based on GBM patients receiving RT/TMZ therapy for recurrent disease includes PS, corticosteroid therapy, number of lesions, and lesion size [29]. As PS is the only consistent factor in all three studies, additional research is needed. Nonetheless, the described prognostic models have the potential to be valuable tools for clinicians when deciding which therapeutic modality is the best for the individual GBM patients.

## Conclusions

This study demonstrates a significant impact of patient age, ECOG PS and status of corticosteroid therapy on TTP and OS for GBM patients treated with RT/TMZ as primary therapy and re-operation or BEV/IRI as secondary therapy. Further by assembling these variables in a model the survival chances at different time-points from diagnosis can be predicted for GBM patients receiving the described therapy.

## Competing interests

The authors declare that they have no competing interests.

## Author’s contributions

SRM and HSP have contributed with the design of the study, interpretation of data and drafting of manuscript. IJC has contributed substantially to the analysis and interpretation of data. KG and HB have contributed by the collection and analysis of data. M-TS and MK have contributed substantially to the manuscript drafting and revision. All authors read and approved the final manuscript.

## Pre-publication history

The pre-publication history for this paper can be accessed here:

http://www.biomedcentral.com/1471-2407/13/402/prepub
